# Enantioselective desymmetrization strategy of prochiral 1,3-diols in natural product synthesis

**DOI:** 10.3762/bjoc.21.151

**Published:** 2025-09-18

**Authors:** Lihua Wei, Rui Yang, Zhifeng Shi, Zhiqiang Ma

**Affiliations:** 1 Key Lab of Functional Molecular Engineering of Guangdong Province, School of Chemistry & Chemical Engineering, South China University of Technology, Guangzhou 510641, P. R. China; 2 Medical Devices Research and Testing Center, South China University of Technology, Guangzhou 510006, P. R. China

**Keywords:** asymmetric synthesis, desymmetrization, 1,3-diols, natural product, total synthesis

## Abstract

Enantioselective desymmetrization is employed as a powerful tool for the creation of chiral centers. Within this scope, the enantioselective desymmetrization of prochiral 1,3-diols, which generates chiral centers by enantioselective functionalization of one hydroxy group, offers beneficial procedures for accessing diverse structural motifs. In this review, we highlight a curated compilation of publications, focusing on the applications of enantioselective desymmetrization of prochiral 1,3-diols in the synthesis of natural products and biologically active molecules. Based on the reaction types, three strategies are discussed: enzymatic acylation, transition-metal-catalyzed acylation, and local desymmetrization.

## Introduction

Natural products isolated from organisms are often asymmetric in their spatial structures, and these unique spatial structures are precisely what lead to their diverse biological activities [1–4]. For the synthesis of these natural products or bioactive molecules, chemists usually need to consider how to carry out asymmetric synthesis of them, driving the advancement of asymmetric methodologies [5–9].

Enantioselective desymmetrization of symmetric substrates has emerged as a pivotal methodology for the construction of chiral centers over the past few decades [10–13]. A series of reaction types have been developed, employing enzymes, metal complexes, or organocatalysts to convert prochiral or *meso* precursors into chiral motifs. Different from other strategies constructing chiral centers by formation of a new chemical bond at the central carbon, enantioselective desymmetrization is achieved through selective reaction at one of the symmetrical functional groups in the precursor, thereby breaking the symmetry and establishing a chiral center. Meanwhile, since the site where the reaction occurs is distant from the newly formed stereocenter, this strategy offers unique advantages, especially in the synthesis of complex molecules which are spatially crowded.

Among various types of substrates for enantioselective desymmetrization, symmetrical diols, especially prochiral 1,3-diols, are often prioritized for testing, because the two primary alcohols of the products (one of them is functionalized in an enantioselective manner) can be utilized for a series of transformations, including functionalization, chain elongation, ring formation, etc. Therefore, the enantioselective desymmetrization of diols has drawn considerable interest among synthetic chemists. Several comprehensive reviews [14–18] on the desymmetrization strategies for diols, including enzymatic desymmetrization and organocatalytic approaches, have been published in the past decade, most of which focus on the methodological development. Although there are reviews on desymmetrization in natural product synthesis [19–21], none of these have put emphasis on the desymmetrization of diols.

Prochiral 1,3-diols, as simple and practical substrates, have been widely used in developed desymmetrization methodologies with applications in the total synthesis of natural products and bioactive molecules, including enzymatic acylation, transition-metal-catalyzed acylation, and local desymmetrization. In this review, we cover total syntheses that utilize enantioselective desymmetrization of prochiral 1,3-diols.

## Review

### Desymmetrization via enzymatic acylation

Enzymatic reactions represent one of the most useful tools in total synthesis. Through combination with organic reactions, this chemo-enzymatic strategy has been successfully utilized in the synthesis of complex molecules [22–23]. Enzymatic reactions feature a convenient operation due to their relative insensitivity to water and oxygen, as well as a specificity to certain substrates, resulting in high enantioselectivity. However, since an enzymatic reaction generally produces only one of the two enantiomers, extensive enzyme screening is often required to access the desired enantiomer.

**Scheme 1:**
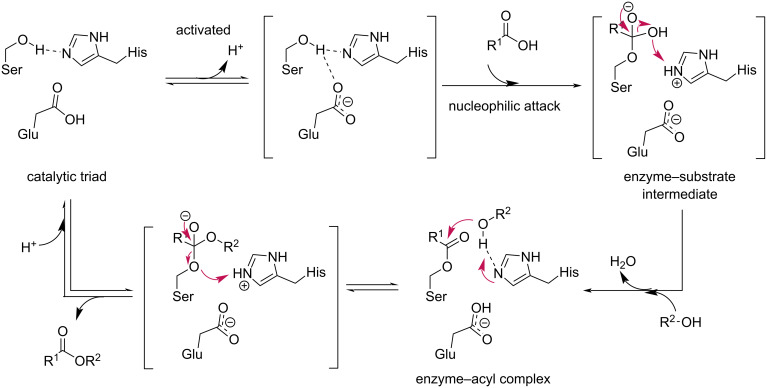
General mechanism of a lipase-catalyzed esterification.

Among various types of enzymes, lipases have proven to be efficient for the desymmetrization of 1,3-diols. Lipases commonly share typical sequences of α-helices and β-strands and possess a catalytic triad consisting of serine (Ser), histidine (His), and aspartate (Asp) or glutamate (Glu). These three amino residues function as a nucleophile-base–acid catalytic system to facilitate esterification, and the general mechanism of a reaction catalyzed by lipases is illustrated in Scheme 1. Additionally, the diverse three-dimensional structures of lipases confer enantioselectivity in lipase-catalyzed esterification [24–25].

Moreover, their commercial availability makes lipases an attractive option for preparing optically pure intermediates in total synthesis. This section focuses on applications of lipase-catalyzed acylation of prochiral 1,3-diols in total synthesis.

#### Porcine pancreatic lipase (PPL)

**Scheme 2:**
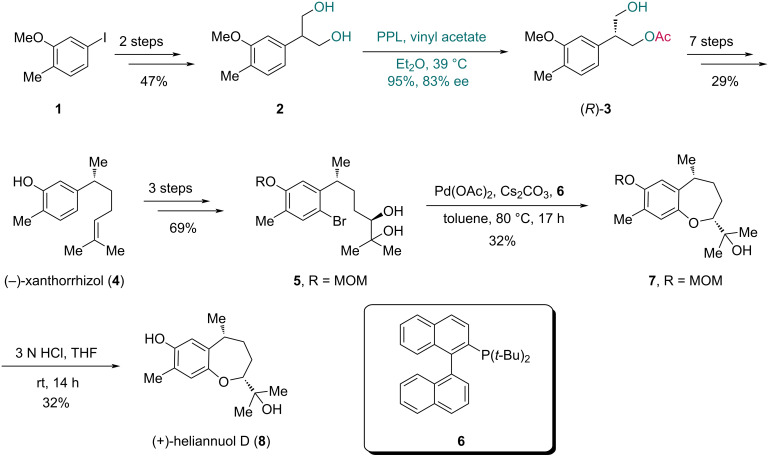
Shishido’s synthesis of (−)-xanthorrhizol (**4**) and (+)-heliannuol D (**8**).

PPL, a commercially available lipase isolated from fresh porcine pancreas [26], is one of the most widely used lipases for asymmetric acylation in total synthesis. In 1999, the Shishido group completed the asymmetric synthesis of (−)-xanthorrhizol, a bioactive bisabolene-type sesquiterpenoid, employing a PPL-catalyzed acylation as the key step (Scheme 2) [27]. The prochiral diol **2** was synthesized from compound **1** in two steps. Subsequently, asymmetric acetylation of **2** catalyzed by PPL afforded (*R*)-**3** in 95% yield with 83% ee. The authors also used *Candida antarctica* lipase (CAL) in this transformation but with a suboptimal result ((*S*)-**3** in 19% yield with 94% ee). The monoacetate (*R*)-**3** was further converted into (−)-xanthorrhizol (**4**) in seven steps. Later in 2003, they further accomplished the synthesis of (+)-heliannuol D, a sesquiterpenoid isolated from sunflower (*Helianthus annuus* L. SH-222), starting from **4** [28]. A three-step sequence transformed **4** into diol **5**. Treatment of **5** with Pd(OAc)_2_ and JohnPhos (**6**) induced cyclization, yielding bicyclic compound **7** with a 7-membered heterocycle. Final deprotection of the methoxymethyl (MOM) group in **7** afforded (+)-heliannuol D (**8**).

**Scheme 3:**
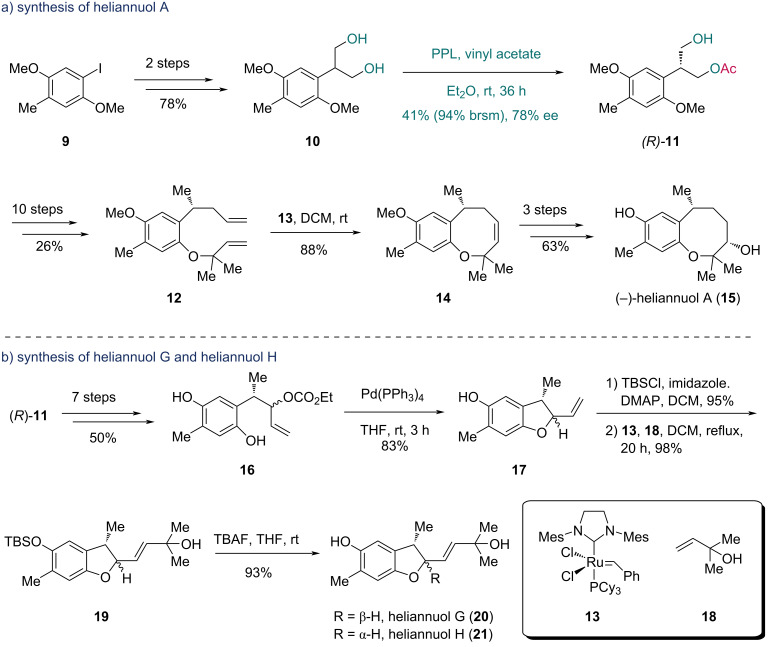
Shishido’s synthesis of a) (−)-heliannuol A (**15**) and b) heliannuol G (**20**) and heliannuol H (**21**).

Having successfully applied PPL-catalyzed acetylation to the synthesis of (+)-heliannuol D, the Shishido group subsequently extended this strategy to other helianane-type sesquiterpenes. In 2003, they completed the enantioselective total synthesis of (−)-heliannuol A, another allelochemical sesquiterpenoid from *Helianthus annuus* L. SH-222 (Scheme 3a) [29]. The aryl iodide **9** was transformed into prochiral diol **10** in two steps. PPL-catalyzed desymmetrization of **10** with vinyl acetate yielded monoacetate (*R*)-**11** in 41% yield (94% brsm) with 78% ee. Diene **12** was prepared from (*R*)-**11** via a ten-step sequence. The following ring-closing metathesis (RCM) reaction catalyzed by Grubbs catalyst **13** converted **12** into the bicyclic compound **14**, which was transformed into (−)-heliannuol A (**15**) in three additional steps.

In 2006, the Shishido group further achieved the synthesis of heliannuol G and heliannuol H (Scheme 3b) [30]. Initially, the authors converted (*R*)-**11** into hydroquinone **16** through a seven-step sequence. The Pd-catalyzed intramolecular cyclization of **16** generated benzofuran **17** in 83% yield. After protecting the phenolic hydroxy group of **17**, cross-metathesis (CM) with allylic alcohol **18** catalyzed by **13** furnished intermediate **19**. Desilylation of **19** produced heliannuol G (**20**) and heliannuol H (**21**), with the structure of **21** confirmed by X-ray crystallographic analysis. Comparative analysis of the ^1^H NMR data with authentic samples of the natural heliannuol G and heliannuol H enabled structural revision of these compounds, correcting prior misassignments in the literature [31–32]. Through enzyme-catalyzed asymmetric acetylation of prochiral 1,3-diols to access chiral building blocks (*R*)-**3** and (*R*)-**11**, Shishido's team completed a series of helianane-type sesquiterpenes. This pioneering work demonstrates the utility of prochiral 1,3-diols in the synthesis of natural products.

**Scheme 4:**
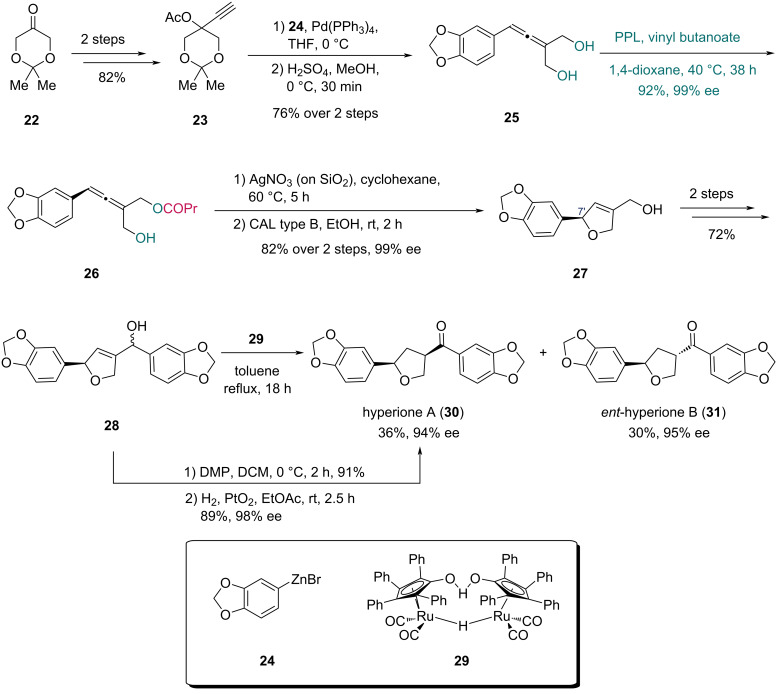
Deska’s synthesis of hyperione A (**30**) and *ent*-hyperione B (**31**).

In 2013, the first asymmetric synthesis of the norlignans hyperione A and *ent*-hyperione B was reported by the Deska group (Scheme 4) [33]. The synthesis commenced with a two-step conversion of ketone **22** to alkyne **23**. Pd-catalyzed Tsuji-type reaction with zinc reagent **24**, followed by acetonide hydrolysis, furnished allenic diol **25**. Treating allenic diol **25** with vinyl butanoate and PPL delivered monoester **26** in 92% yield (99% ee). The axial chirality was transferred to the C7’ stereocenter through a Ag(I)-catalyzed cycloisomerization of the allenol, constructing the dihydrofuran ring. Lipase-catalyzed ester hydrolysis provided allylic alcohol **27**. Alcohol **28** was obtained from **27** in two steps, and was subsequently converted to hyperione A (**30**) and *ent*-hyperione B (**31**) by refluxing in toluene with Shvo’s catalyst **29**. Notably, the authors found that hyperione A (**30**) could be obtained in higher yield and enantiopurity from alcohol **28** via a two-step sequence including oxidation and subsequent hydrogenation.

**Scheme 5:**
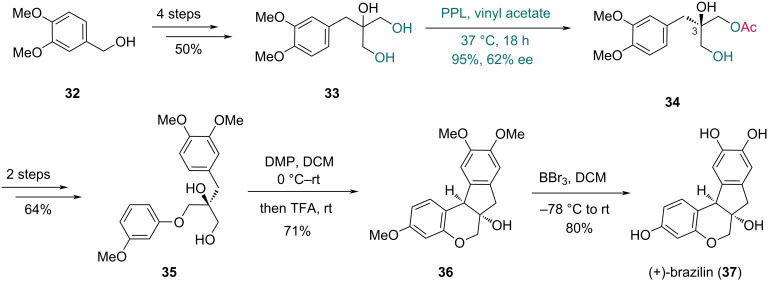
Huang’s synthesis of (+)-brazilin (**37**).

The Huang group reported their synthesis of (+)-brazilin and its racemic form in 2022 (Scheme 5) [34]. They first evaluated the feasibility of the Prins/Friedel–Crafts tandem reaction in the construction of the 6/6/5/6 tetracyclic skeleton, successfully completing the racemic synthesis of brazilin. For the asymmetric synthesis, the C3 chiral center of (+)-brazilin was established via enzymatic desymmetrization. Triol **33** was prepared from alcohol **32** in four steps. PPL-catalyzed desymmetrization of **33** afforded chiral monoester **34** in 95% yield with 62% ee. A two-step conversion of **34** gave diol **35**, which underwent Prins/Friedel–Crafts tandem cyclization to construct tetracyclic compound **36**. Final deprotection delivered (+)-brazilin (**37**).

#### *Candida antarctica* lipase (CAL)

**Scheme 6:**
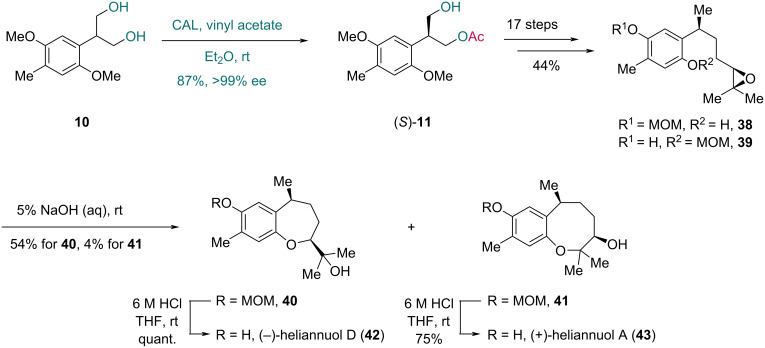
Shishido’s synthesis of (−)-heliannuol D (**42**) and (+)-heliannuol A (**43**).

CAL is a type of lipase originating from the yeast *Candida antarctica* and includes two enzymes, CAL-A and CAL-B [35–36]. Although a previous report [27] indicated that the desymmetrization of prochiral diol **2** with CAL was ineffective, the Shishido group prepared optically active compound (*S*)-**11** via CAL-catalyzed asymmetric transesterification of the structurally similar diol **10**, thus completing the enantioselective synthesis of (−)-heliannuol D and (+)-heliannuol A (Scheme 6) [37]. The monoester (*S*)-**11** was isolated in 87% yield with >99% ee. A subsequent 17-step sequence provided epoxides **38** and **39**. Treatment of the mixture of **38** and **39** with 5% NaOH aqueous solution resulted in intramolecular [7-*exo*] and [8-*endo*] cyclization, furnishing the 7-membered cyclic ether **40** and 8-membered cyclic ether **41**, respectively. Finally, MOM deprotection produced (−)-heliannuol D (**42**) and (+)-heliannuol A (**43**).

**Scheme 7:**
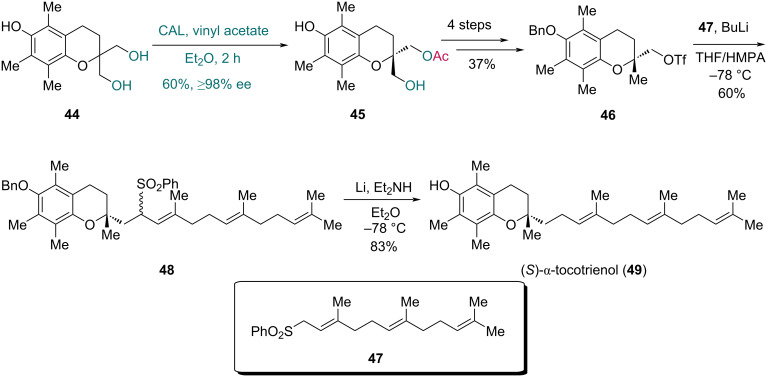
Chênevert’s synthesis of (*S*)-α-tocotrienol (**49**).

In 2002, Chênevert and co-workers completed the total synthesis of (*S*)-α-tocotrienol, a natural isoform of vitamin E (Scheme 7) [38]. The authors used known triol **44** as the starting material. In the desymmetrization promoted by CAL, triol **44** underwent a monoacetylation process, providing chiral compound **45** in 60% yield with over 98% ee. After a four-step conversion of **45** to triflate **46**, alkylation with sulfone **47** via treatment with butyllithium and hexamethylphosphoramide (HMPA) yielded the coupling product **48** as a mixture of diastereoisomers in 60% yield. Ultimately, single-electron reduction removed both the sulfone and benzyl groups of **48**, furnishing (*S*)-α-tocotrienol (**49**) in 83% yield.

#### *Candida rugosa* lipase (CRL)

**Scheme 8:**
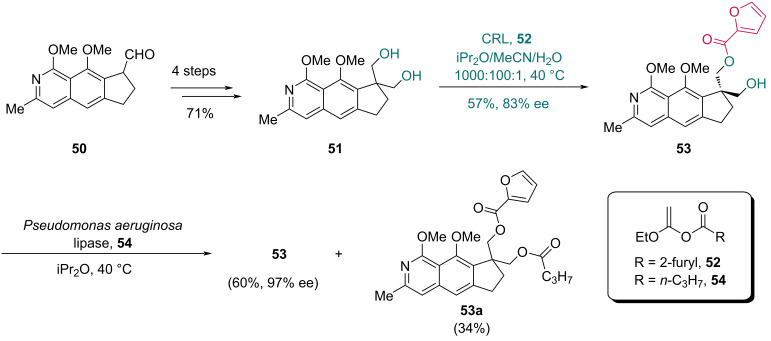
Kita’s synthesis of monoester **53**.

The lipase CRL from *Candida rugosa,* another species of *Candida* genus*,* was used by the Kita group in their asymmetric synthesis of fredericamycin A in 2005 (Scheme 8) [39]. Different from their previous strategy [40] constructing the spiro chiral center via Lewis acid-mediated semi-pinacol rearrangement, this work involved a CRL-catalyzed desymmetrization of prochiral diol **51** (prepared from aldehyde **50** in four steps), providing monoester **53** in 57% yield with 83% ee. Notably, 1-ethoxyvinyl 2-furoate (**52**) was selected as the acyl donor in this step to suppress potential intramolecular acyl migration. To further improve the optical purity of monoester **53**, a *Pseudomonas aeruginosa* lipase-mediated kinetic resolution was performed with ethoxyvinyl butyrate **54**, ultimately achieving monoester **53** with 97% ee in 60% yield and the diester **53a**.

**Scheme 9:**
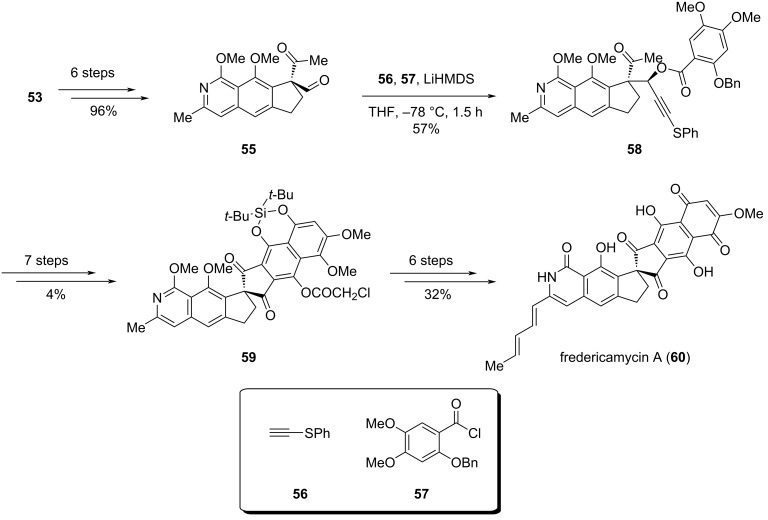
Kita’s synthesis of fredericamycin A (**60**).

With enantioenriched monoester **53** in hand, the synthesis proceeded toward fredericamycin A (**60**) (Scheme 9). Dione **55**, which was prepared from **53** in six steps, underwent addition with alkyne **56** followed by acylation of the resulting hydroxy group with compound **57** to yield ketone **58**. A subsequent seven-step transformation involving acyl-group migration, [4 + 2] cycloaddition and aromatic Pummerer-type reaction, provided chiral spiro compound **59** with the 6/6/5/5/6/6 scaffold, and this intermediate was further elaborated to **60** in six additional steps.

#### Lipases from *Pseudomonas* genus

**Scheme 10:**
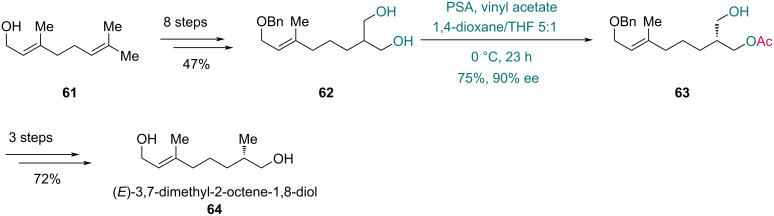
Takabe’s synthesis of (*E*)-3,7-dimethyl-2-octene-1,8-diol (**64**).

*Pseudomonas* is a genus of Gram-negative bacteria widely distributed in nature [41]. Some species within this genus produce lipases that effectively catalyze the desymmetrization of prochiral diols, which were used in total syntheses. In 2003, an enzymatic asymmetric acylation with PSA, a lipase from *Pseudomonas cepacia*, was adopted by Takabe and co-workers in their synthesis of (*E*)-3,7-dimethyl-2-octene-1,8-diol (isolated from *Danaus chrysippus*) (Scheme 10) [42]. Prepared from geraniol (**61**) in eight steps, diol **62** was converted to enantioenriched compound **63** in 75% yield with 90% ee in the presence of PSA. This intermediate was further advanced to (*E*)-3,7-dimethyl-2-octene-1,8-diol (**64**) over three steps.

**Scheme 11:**
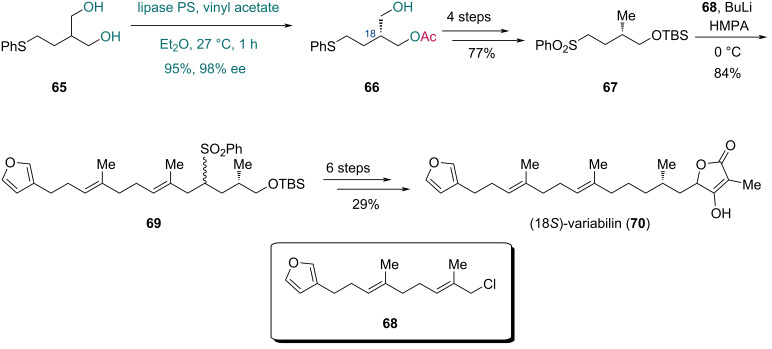
Takabe’s synthesis of (18*S*)-variabilin (**70**).

Later in 2004, Takabe and co-workers accomplished the asymmetric synthesis of variabilin, a marine-derived furanosesterterpene (Scheme 11) [43]. The key C18 chiral center was established through lipase-mediated asymmetric transesterification. After substrates screening, diol **65** was selected and converted into monoester **66** in 95% yield with 98% ee using vinyl acetate and lipase PS from *Pseudomonas cepacia*. Four subsequent steps afforded sulfone **67**, and the following alkylation with fragment **68** in the presence of butyllithium and HMPA produced coupling product **69** in 84% yield. Finally, a six-step sequence completed the synthesis of (18*S*)-variabilin (**70**).

**Scheme 12:**
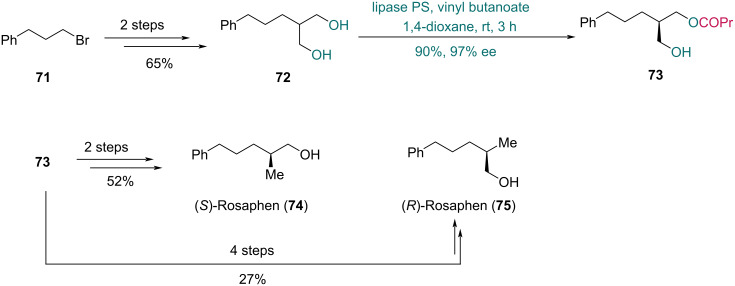
Kawasaki’s synthesis of (*S*)-Rosaphen (**74**) and (*R*)-Rosaphen (**75**).

In 2010, Kawasaki and co-workers reported the asymmetric synthesis of both (*S*)-Rosaphen and (*R*)-Rosaphen to evaluate their odor profiles (Scheme 12) [44]. Diol **72** was prepared from bromide **71** in two steps. Lipase PS-mediated desymmetrization of **72** with vinyl butanoate provided monoester **73** in 90% yield with 97% ee. To obtain (*S*)-Rosaphen (**74**), monoester **73** was converted via mesylation followed by hydride reduction. In contrast, the synthesis of (*R*)-Rosaphen (**75**) required a four-step sequence comprising TBS protection, ester hydrolysis, mesylation, and hydride reduction.

**Scheme 13:**
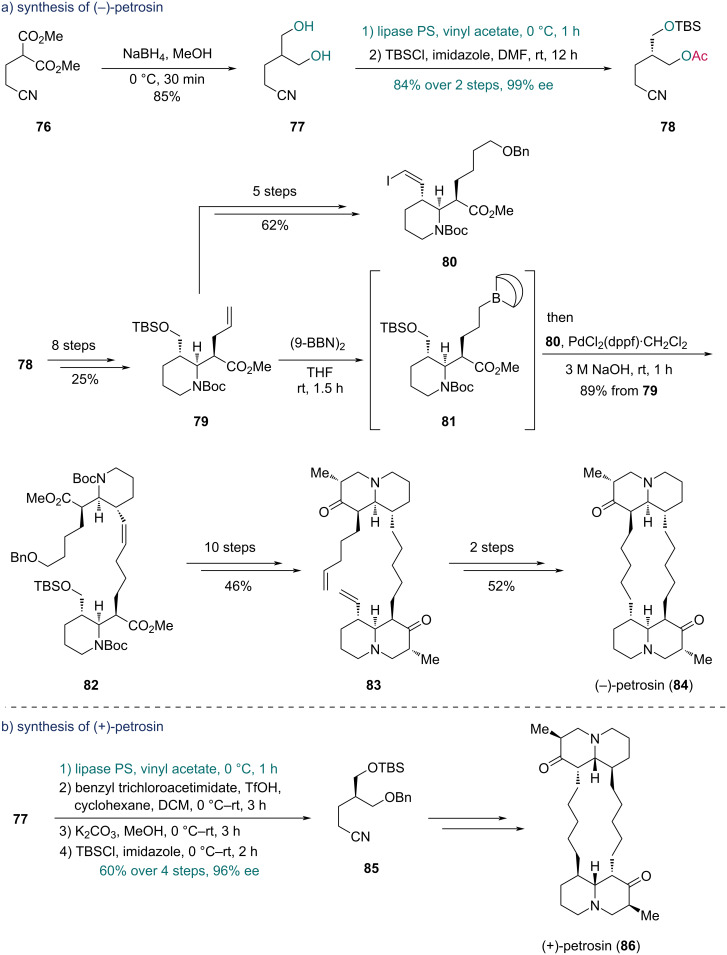
Tokuyama’s synthesis of a) (−)-petrosin (**84**) and b) (+)-petrosin (**86**).

In 2014, Tokuyama and co-workers accomplished the total synthesis of (−)-petrosin and (+)-petrosin, two marine-derived bisquinolizidine alkaloids [45]. They first completed the synthesis of (−)-petrosin (**84**) (Scheme 13a). Prochiral diol **77**, produced from diester **76** through reduction, was subjected to a lipase PS-mediated asymmetric transesterification. The resulting enantioenriched monoester, on hydroxy group protection with *tert*-butyldimethylsilyl chloride (TBSCl), yielded compound **78** in 84% yield over two steps with 99% ee. The TBS protection was crucial to prevent the potential racemization by intramolecular transesterification. Ester **79** was then prepared from **78** in eight steps. To complete the dimerization, fragments **80** and **81** were independently prepared from **79**. An intermolecular Suzuki–Miyaura coupling between **80** and **81** gave diester **82**. Through a ten-step sequence including an aza-Michael reaction, diester **82** was converted into diketone **83**, which was further transformed into (−)-petrosin (**84**) via RCM reaction and hydrogenation. For the synthesis of (+)-petrosin (**86**) (Scheme 13b), a similar strategy was adopted using compound **85** as the synthetic intermediate, which was prepared from diol **77** in a four-step sequence with 60% overall yield and 96% ee.

**Scheme 14:**
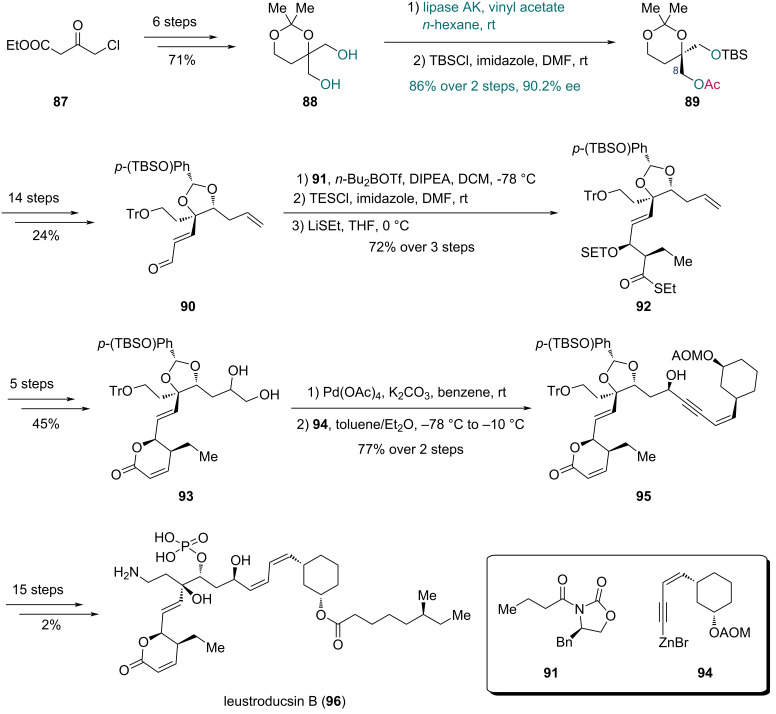
Fukuyama’s synthesis of leustroducsin B (**96**).

In 2003, the Fukuyama group realized the first total synthesis of leustroducsin B, a microbial metabolite with various biological activities, featuring a lipase AK (from *Pseudomonas fluorescens*)-mediated desymmetrization (Scheme 14) [46]. Starting with known compound **87**, the prochiral diol **88** was prepared in six steps. Subsequent asymmetric transesterification in the presence of vinyl acetate and lipase AK afforded the optically active acetate, which was followed by TBS protection of the free hydroxy group to give compound **89**, establishing the C8 chiral center in 86% yield over two steps with 90% ee. A further 14-step sequence furnished enone **90**, which underwent Evans aldol reaction with fragment **91**. After triethylsilyl (TES) protection of the resulting hydroxy group and auxiliary cleavage, thioester **92** was obtained. Five additional steps converted **92** into lactone **93**. Oxidative cleavage of the diol group in **93** and following coupling with fragment **94** gave compound **95**, which was further elaborated to leustroducsin B (**96**) in 15 steps.

**Scheme 15:**
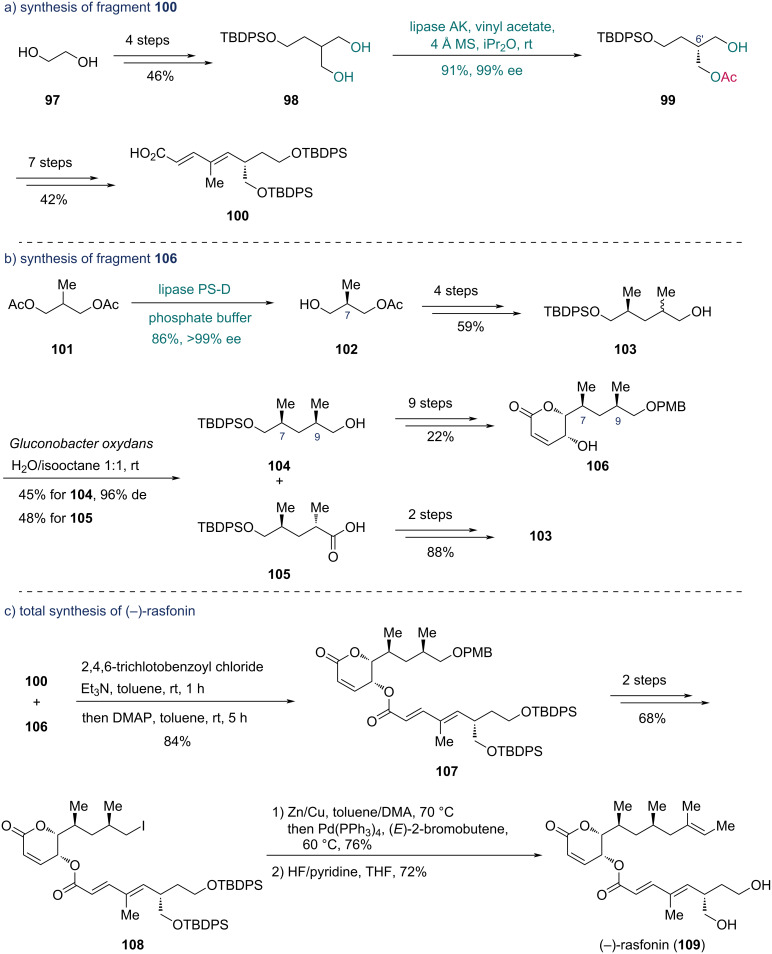
Nanda’s synthesis of a) fragment **100**, b) fragment **106** and c) (−)-rasfonin (**109**).

In 2013, Nanda and co-worker described the asymmetric synthesis of (−)-rasfonin, harnessing an enantioselective enzymatic desymmetrization with lipase AK and an enzymatic oxidative kinetic resolution to install stereocenters [47]. The synthesis commenced with the preparation of fragment **100** from ethylene glycol (**97**) (Scheme 15a). Through a four-step sequence, diol **98** was prepared from **97**, which underwent enzymatic desymmetrization with lipase AK in the presence of vinyl acetate to yield monoacetate **99** in 91% yield and 99% ee. This transformation established the C6’ chiral center. Seven additional steps enabled the synthesis of fragment **100**. For the synthesis of fragment **106** (Scheme 15b), enzymatic hydrolysis of racemic diacetate **101** catalyzed by lipase PS-D (from *Pseudomonas cepacia*, immobilized on diatomite) was performed to deliver monoacetate **102** with the desired C7 chiral center in >99% ee. After four steps of functional group manipulations, alcohol **103** was subjected to enzymatic oxidative kinetic resolution with the bacterium *Gluconobacter oxydans*, producing alcohol **104** and acid **105**. The alcohol **104** with the desired C9 stereocenter was then converted into fragment **106** in nine steps, while acid **105** was recycled to **103** in two steps.

With the fragments **100** and **106** in hand, the synthesis of (−)-rasfonin proceeded via Yamaguchi esterification between the two fragments to obtain lactone **107** (Scheme 15c). A subsequent two-step transformation yielded compound **108**, which underwent Stille coupling with (*E*)-2-bromobutene followed by desilylation to afford (−)-rasfonin (**109**).

**Scheme 16:**
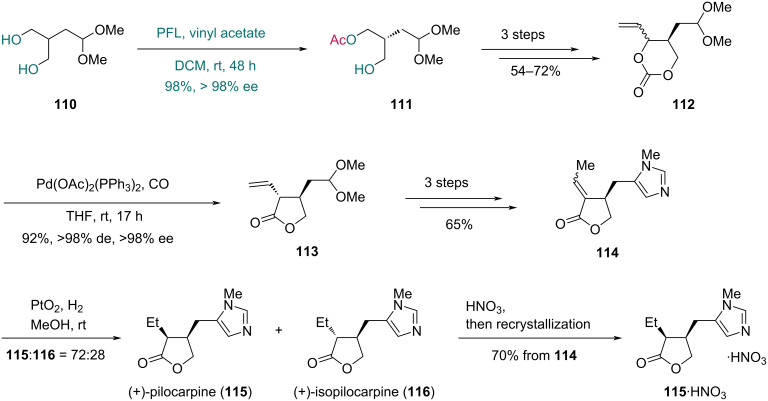
Davies’ synthesis of (+)-pilocarpine (**115**) and (+)-isopilocarpine (**116**).

In 2009, Davies and co-workers disclosed the asymmetric synthesis of (+)-pilocarpine and (+)-isopilocarpine using an enzyme-catalyzed acetylation with *Pseudomonas fluorescens* lipase (PFL) (Scheme 16) [48]. Treatment of diol **110** with PFL and vinyl acetate gave monoacetate **111** in 98% yield and >98% ee. Subsequently, monoacetate **111** was converted into compound **112** with a 1,3-dioxan-2-one moiety in three steps, which underwent Pd-catalyzed decarboxylation/carbonylation to form the lactone **113**. The *N*-methylimidazole ring was installed through a three-step sequence to give lactone **114**. Finally, hydrogenation of **114** provided (+)-pilocarpine (**115**) and (+)-isopilocarpine (**116**) in a ratio of 72:28. Treatment of the mixture with HNO_3_ followed by recrystallization afforded the nitrate salt of **115** (**115**·HNO_3_) in 70% yield from **114**.

**Scheme 17:**
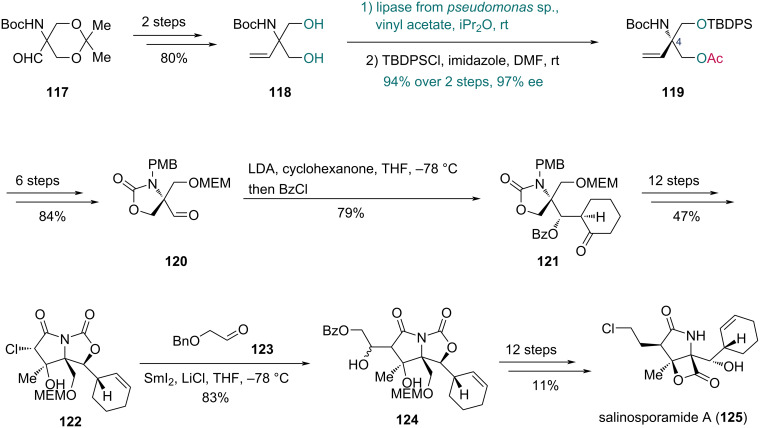
Ōmura’s synthesis of salinosporamide A (**125**).

In 2008, the Ōmura group completed the total synthesis of salinosporamide A, a marine-derived natural product with anticancer activity, featuring an enzymatic desymmetrization (Scheme 17) [49]. To establish the C4 chiral center, prochiral diol **118** (prepared from known compound **117**) was treated with lipase from *Pseudomonas* sp. (WAKO) and vinyl acetate, affording the corresponding monoacetate. Subsequent reaction with *tert*-butyldiphenylsilyl chloride (TBDPSCl) and imidazole provided compound **119** in 94% yield over two steps with 97% ee. Next, compound **120** was obtained in six steps from **119**. A stereoselective aldol reaction installed the cyclohexanone ring into **120**, and the resulting hydroxy group was protected to give ketone **121**. The γ-lactam moiety of compound **122** was then constructed in subsequent 12 steps. SmI_2_-mediated intermolecular Reformatsky-type reaction with aldehyde **123** yielded compound **124**. Finally, salinosporamide A (**125**) was obtained through a 12-step sequence from **124**.

### Desymmetrization via transition-metal-catalyzed acylation

Although enzymatic acylation reactions are widely employed in total synthesis, certain substrates are incompatible with acylation catalyzed by existing lipases. Inspired by enzymatic reactions, chemists have developed a series of catalysts composed of transition-metal cores and chiral ligands, which have been applied to various asymmetric reactions [50–52]. Compared to the enzymatic methods, the transition-metal-catalyzed approach may provide an advantage to access both enantiomers of the product in the same process by employing the antipodal ligand, as both enantiomers of the chiral ligand are normally accessible. Additionally, the substrate scope can be broadened by modifying the ligand’s structure.

Early in 1984, Ichikawa and co-workers reported a Sn-mediated enantioselective acylation of glycerol derivatives [53]. Since then, desymmetrization strategies for prochiral 1,3-diols involving transition-metal-catalyzed acylation have been developed. Trost and co-workers then developed a Zn-based catalyst for asymmetric aldol reactions [54–55], later adapting it to the desymmetrization of 1,3-diols in 2003 [56]. Subsequent advances included Cu-based complexes developed by Kang and co-workers [57–58], first applied in total synthesis in 2008. In this section, examples of transition-metal-catalyzed acylations of prochiral 1,3-diols in total synthesis are discussed, including Cu-catalyzed and Zn-catalyzed acylation reactions.

#### Cu-catalyzed acylation

**Scheme 18:**
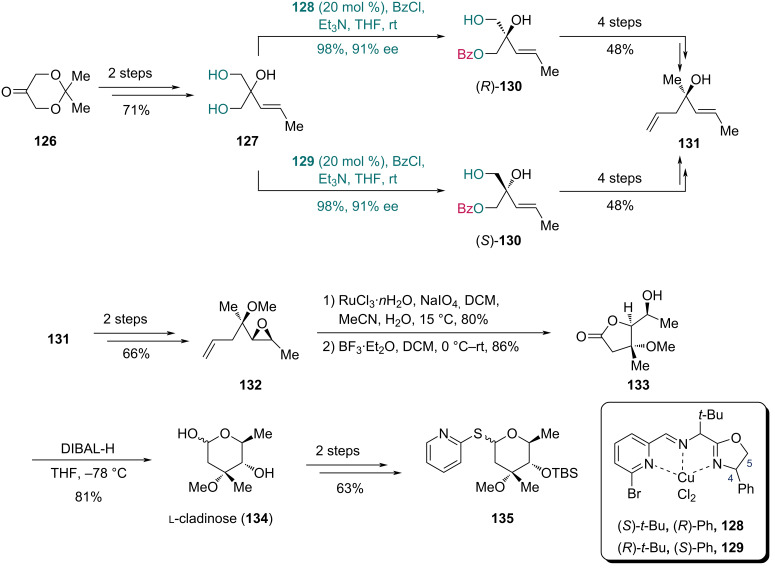
Kang’s synthesis of ʟ-cladinose (**124**) and its derivative.

In 2008, Kang and co-workers demonstrated the first use of Cu-catalyzed enantioselective acylation [57–58] in the synthesis of ʟ-cladinose (Scheme 18) [59]. In the presence of catalyst **128**, triol **127**, prepared from compound **126** in two steps, was converted into (*R*)-**130** with 98% yield and 91% ee, which was subjected to a four-step sequence to give compound **131**. In this reaction, catalyst **128** proved most effective. As previously reported [57], installing a sterically demanding or electronically influential group on the pyridine moiety enhanced the reaction performance. However, excessively bulky substituents at C4 and substitutions at both C4 and C5 hindered the coordination between substrate and catalyst, and led to reduced enantioselectivity. As to the structure of **128**, the electronic effect of the bromo-substituted pyridine moiety favored complexation, while the phenyl substitution at C4 promoted a stable coordination-bond formation. Alternatively, (*S*)-**130** could be furnished using Cu complex **129** in the desymmetrization step with comparable efficiency (98% yield and 91% ee), and was likewise transformed into **131** in four steps. Epoxidation of **131** followed by methylation generated epoxide **132**. Construction of the lactone moiety commenced with the oxidative cleavage of the double bond, and the resulting carboxylic acid underwent intramolecular cyclization in the presence of BF_3_·Et_2_O to give lactone **133**. Subsequent hydride reduction induced rearrangement of **133** to form the pyranose skeleton of ʟ-cladinose (**134**). Finally, the derivative, thiocladinoside **135** was then prepared from **134** in two additional steps.

**Scheme 19:**
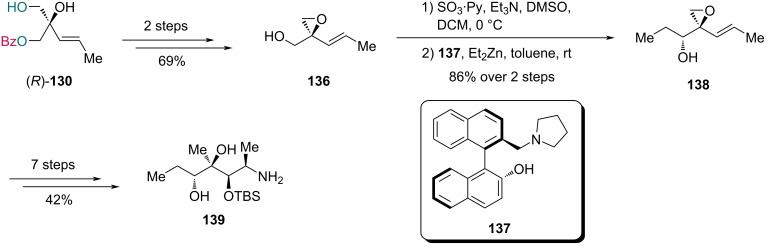
Kang’s preparation of fragment **139**.

The total synthesis of azithromycin [60] was reported shortly after completion of **135** (Scheme 19). For the synthesis of fragment **139**, epoxide **136** was first prepared from (*R*)-**130** in two steps. Parikh–Doering oxidation of **136** followed by addition with Et_2_Zn in the presence of ligand **137** afforded alcohol **138**, which was subsequently converted into amine **139** via a seven-step sequence.

**Scheme 20:**
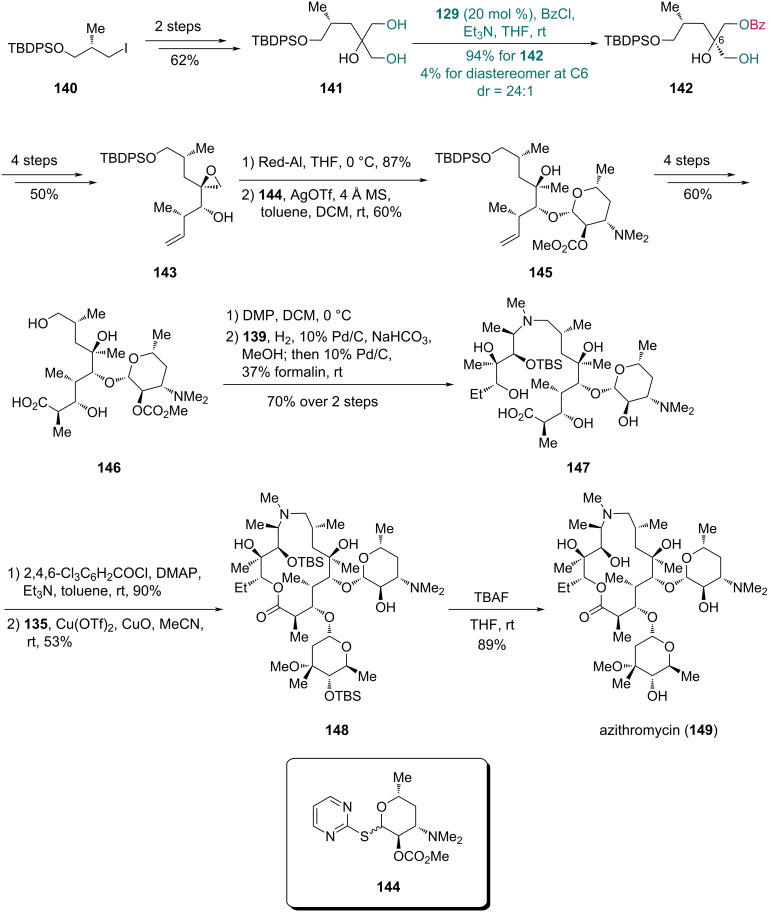
Kang’s synthesis of azithromycin (**149**).

With the fragments **135** and **139** in hand, synthesis of the third fragment **146** was then pursued and further elaborated to complete the synthesis of azithromycin (Scheme 20). Triol **141** was first prepared in two steps from iodide **140**. Subsequent Cu-catalyzed desymmetrization with catalyst **129**, benzoyl chloride (BzCl) and Et_3_N, enabled the synthesis of monobenzoate **142** in 94% yield along with 4% yield of its diastereomer (dr = 24:1). Following a four-step conversion of **142** to epoxide **143**, reductive cleavage produced a diol intermediate, which was subjected to chemoselective glycosylation with compound **144** to provide compound **145**. After a four-step transformation of **145**, compound **146** was oxidized with Dess-Martin periodinane (DMP). Subsequent reductive amination with fragment **139** provided an intermediate, which underwent the second reductive amination using formaldehyde. This one-pot process with concomitant deprotection afforded acid **147** in 70% yield over two steps. Macrocyclization of **147**, followed by glycosylation with **135**, gave compound **148**, which was converted into azithromycin (**149**) upon desilylation.

**Scheme 21:**
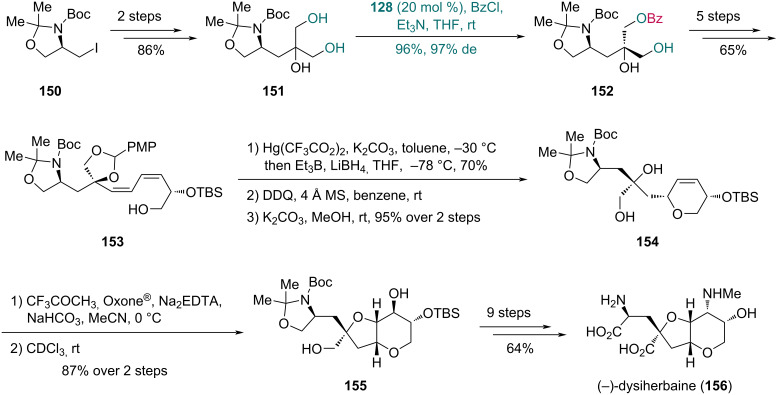
Kang’s synthesis of (−)-dysiherbaine (**156**).

This desymmetrization strategy was also employed in the synthesis of (−)-dysiherbaine reported by Kang and co-workers in 2012 (Scheme 21) [61]. Their synthesis commenced with compound **150**, which was converted into triol **151** in two steps. Treatment of triol **151** with catalyst **128** furnished monobenzoate **152** in 96% yield and 97% de*.* Subsequently, monobenzoate **152** was transformed into diene **153** in five steps. The *cis*-3,6-disubstituted dihydropyran ring was assembled via a one-pot mercuriocyclization/reductive demercuration of **153** followed by two-step diol-deprotection to access compound **154**. Using trifluoromethylmethyldioxirane, which was generated in situ from trifluoroacetone, Oxone^®^, and disodium ethylenediaminetetraacetate dihydrate (Na_2_EDTA), compound **154** underwent epoxidation followed by acid-mediated cyclization to yield bicyclic compound **155**. The synthesis was completed through a nine-step conversion of **155** to obtain (−)-dysiherbaine (**156**).

**Scheme 22:**
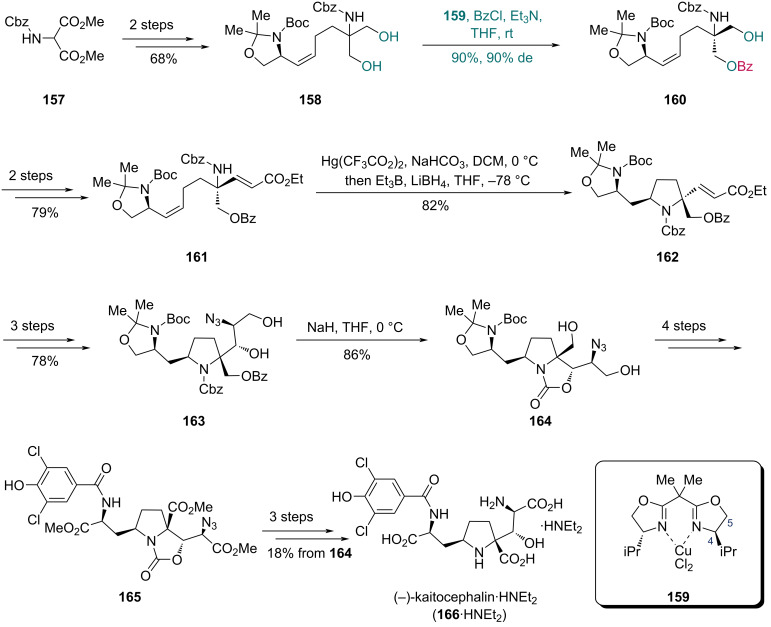
Kang’s synthesis of (−)-kaitocephalin (**166**).

To construct the asymmetric quaternary carbon centers with an amino group, the Kang group developed a desymmetrization strategy for serinol derivatives using a bisoxazoline (BOX)–CuCl_2_ complex as catalyst in 2008 [62]. They further applied this method in 2013 to the synthesis of (−)-kaitocephalin, a glutamate receptor antagonist from *Eupenicillium shearii* (Scheme 22) [63]. Diol **158**, which was accessed in two steps from diester **157**, underwent enantioselective monobenzoylation with complex **159** as catalyst to form benzoate **160** in 90% yield with 90% de. The size of the C4-substituent in the oxazoline moiety crucially influenced the enantioselectivity and conversion of the reaction: smaller substituents reduced the differential ability between two hydroxy groups, while bulky substituents hindered the formation of coordination bonds between the substrate and catalyst. As previously reported, with a suitable substituent at C4, an additional C5-substituent slightly enhanced the catalytic performance of the complex [62]. For diol **158**, the ligand with only isopropyl substitution at C4 proved effective with suitable size for the substrate–catalyst coordination. A subsequent two-step sequence enabled the sythesis of olefinic carbamate **161** from benzoate **160**. Treatment of **161** with Hg(CF_3_CO_2_)_2_ induced mercuriocyclization, followed by reductive demercuration with LiBH_4_/Et_3_B to construct the pyrrolidine ring of compound **162**. A three-step transformation of **162** yielded compound **163**, which was subjected to base-mediated cyclization with concomitant debenzoylation to deliver oxazolidinone **164**. Through a four-step sequence, oxazolidinone **164** was then converted into triester **165**, which was further transformed into (−)-kaitocephalin (**166**) as its diethylamine salt in three additional steps.

**Scheme 23:**
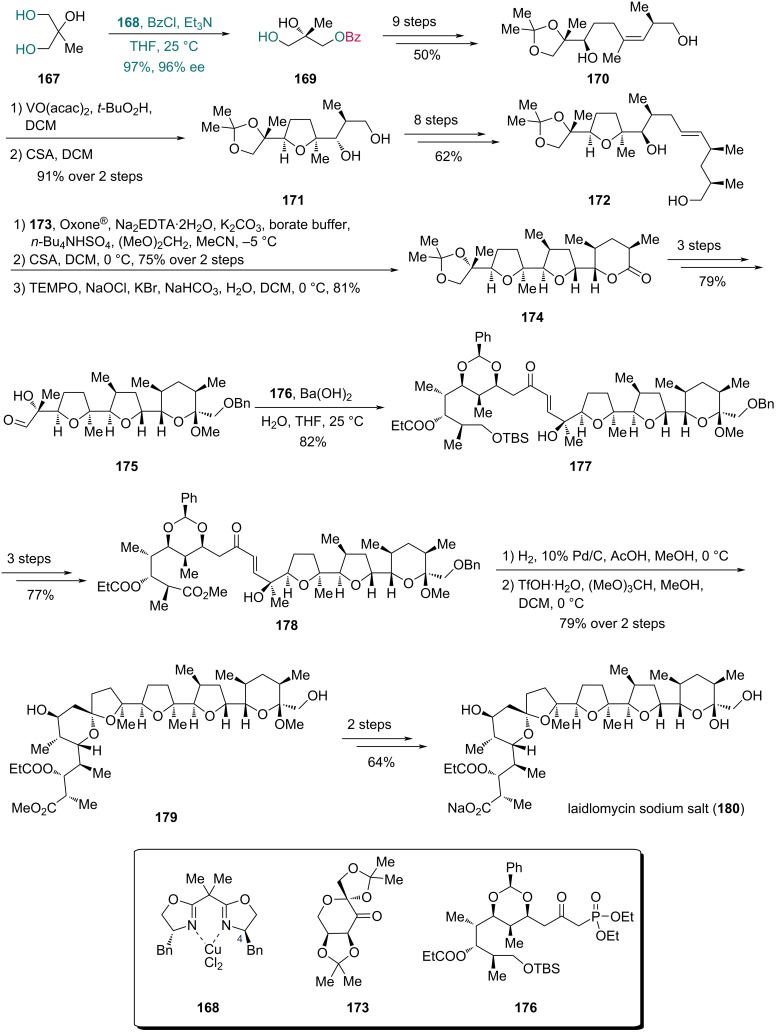
Kang’s synthesis of laidlomycin (**180**).

In Kang’s synthesis of laidlomycin in 2016 (Scheme 23) [64], the BOX–CuCl_2_ complex **168** effectively catalyzed the desymmetrization of triol **167**, affording monobenzoate **169** in 97% yield with 96% ee. For 2-alkyl-substituted glycerols like triol **167**, complex **168** is the most efficient catalyst as the BOX ligand with a benzyl substitution at C4 provided an appropriate size for the catalyst–substrate coordination [58]. The intermediate **169** was transformed into alcohol **170** in nine steps. Subsequent epoxidation of olefin in **170** followed by acid-mediated cyclization provided compound **171** bearing a tetrahydrofuran ring. An eight-step transformation then yielded compound **172**. Next, epoxidation of olefin of **172** with Shi’s dioxirane (generated from ketone **173**) and the following acid-mediated cyclization formed another tetrahydrofuran ring. The resulting compound was then converted into lactone **174** via 2,2,6,6-tetramethylpiperidin-1-oxyl (TEMPO)-mediated oxidation. Lactone **174** was then converted into aldehyde **175** in three steps, which underwent Horner–Wadsworth–Emmons (HWE) olefination with β-ketophosphonate **176** to produce *trans*-enone **177** as the sole product. Ester **178**, prepared in three steps from **177**, first underwent cyclization via hydrogenation to generate spiroketals as a 1:1 mixture. This intermediate was then isomerized under acidic conditions to the desired spiroketal **179**, which was ultimately converted into laidlomycin sodium salt (**180**) in two additional steps.

**Scheme 24:**
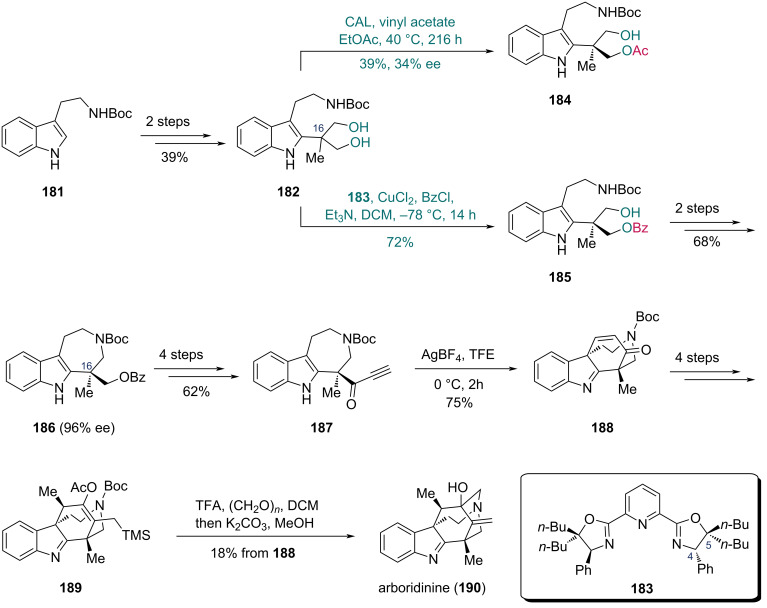
Snyder’s synthesis of arboridinine (**190**).

In 2011, the Kang group developed an enantioselective desymmetrization strategy for 2,2-disubstituted 1,3-propanediols catalyzed by a pyridinebisoxazoline (PyBOX)–CuCl_2_ complex [65]. Snyder and co-workers applied this method to synthesize arboridinine, an indole alkaloid isolated from a Malaysian *Kopsia* species (Scheme 24) [66]. The synthesis commenced with *tert*-butyloxycarbonyl (Boc)-protected tryptamine **181**, which was converted into diol **182** in two steps. Initial attempts to forge the chiral center at C16 via enzyme-catalyzed monoacylation proved unsatisfactory and provided a low yield and ee (39% and 34%, respectively). In contrast, a CuCl_2_ complex bearing a PyBOX-derived ligand **183** effectively catalyzed the desymmetrization of **182**, giving benzoate **185** in 72% yield. The C5-subsituents of ligand **183** are important to adjust the conformation of the ligand to provide suitable space for the smaller group. It is observed that the attachment of two *n*-butyl groups at the C5 position is beneficial for the reaction [65]. Although the ee of monobenzoate **185** was undetermined, azepinoindole **186** prepared in two steps from **185** exhibited 96% ee, indicating high enantioselectivity in the desymmetrization step. A four-step sequence was adopted to convert **186** into ynone **187**, which underwent a Ag-mediated 6-*endo*-*dig* cyclization in trifluoroethanol (TFE) to produce enone **188** containing the tetracyclic core of arboridine. In the presence of trifluoroacetic acid (TFA) and paraformaldehyde, compound **189**, prepared from **188** in four steps, underwent aza-Prins cyclization to form the caged skeleton, and the following acetate hydrolysis afforded arboridinine (**190**) in 38% yield over two steps.

**Scheme 25:**
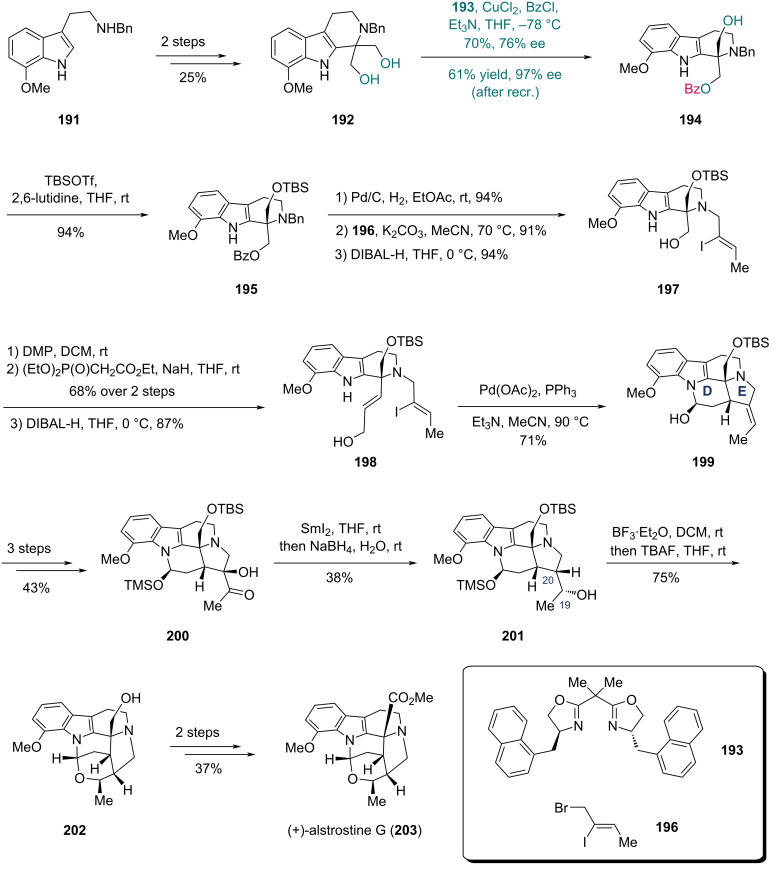
Ma’s synthesis of (+)-alstrostine G (**203**).

In 2024, Ma and co-workers accomplished their synthesis of (+)-alstrostine G with a Cu-catalyzed asymmetric desymmetrization as the key step (Scheme 25) [67]. Diol **192** with a 1,1-disubstituted tetrahydro-β-carboline (THBC) core was prepared from tryptamine derivative **191** via a two-step sequence comprising a Pictet–Spengler reaction followed by reduction. Screening of enantioselective monobenzoylation conditions revealed that using a Cu-based complex composed of 4-(1-naphthylbenzyl)-substituted BOX ligand **193** and CuCl_2_ with Et_3_N and BzCl in THF solution afforded optimal results in terms of both isolated yield and ee. Under these optimized conditions, diol **192** was transformed into monobenzoate **194** in 70% yield with 76% ee, and further recrystallization enhanced the enantiopurity to 97% ee with 61% yield. TBS protection of the hydroxy group in **194** afforded compound **195**. A three-step sequence comprising removal of the benzyl group, chemoselective *N*-alkylation with fragment **196**, and removal of the benzoyl group allowed the conversion of **195** into iodide **197**. Sequential oxidation of the alcohol, HWE reaction, and reduction of the resulting ester then provided compound **198**. In the presence of Pd(OAc)_2_, PPh_3_, and Et_3_N in MeCN, the intramolecular Heck/hemiamination cascade reaction of **198** delivered the 5-*exo* cyclization product **199**, simultaneously constructing the fused D and E rings in a single transformation. Three additional steps converted **199** to hydroxy ketone **200**, which underwent SmI_2_-mediated deoxygenation of **200** and ketone reduction to give compound **201**. Stereoselective hemiaminal ether formation promoted by BF_3_·Et_2_O with subsequent desilylation constructed the hexacyclic framework of alstrostine G, yielding compound **202**. Finally, (+)-alstrostine G (**203**) was obtained through a two-step sequence.

#### Zn-catalyzed acylation

**Scheme 26:**
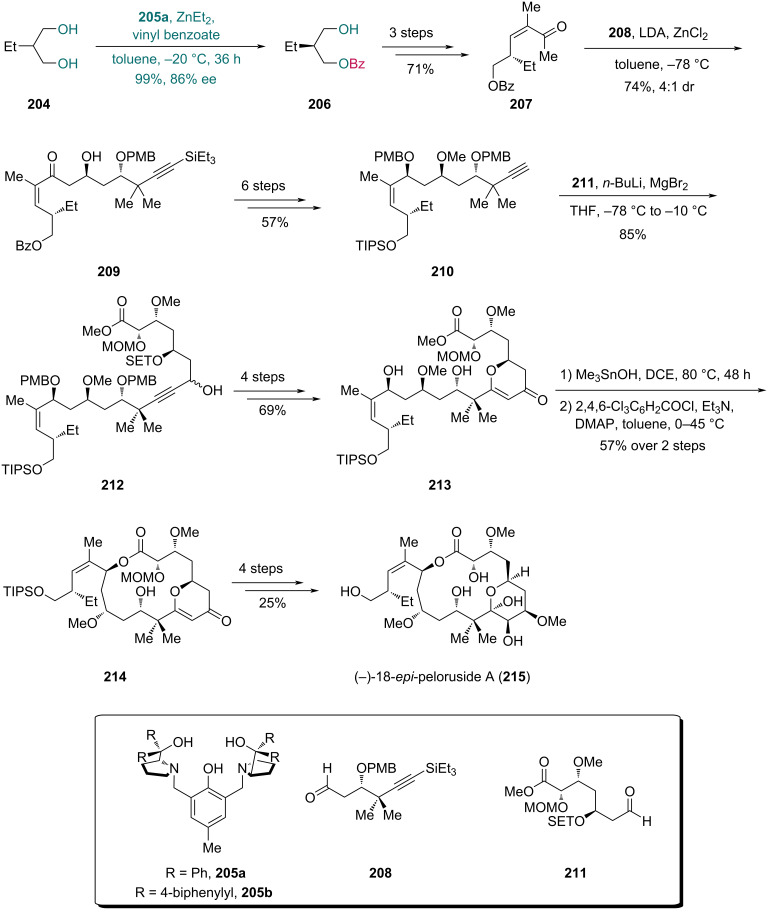
Trost’s synthesis of (−)-18-*epi*-peloruside A (**215**).

Zn-based complexes are another class of effective catalysts used in desymmetrization of 1,3-diols, as reported by Trost and co-worker in 2003 [56]. In 2013, Trost et al. developed the synthesis of (−)-18-*epi*-peloruside A (Scheme 26) [68], and converted diol **204** into enantioenriched monobenzoate **206** using a catalyst composed of ZnEt_2_ and ligand **205a**, affording the product in 99% yield and 86% ee. Although in their previous report [56], the ligand **205b** with a 4-biphenylyl substitution was more efficient than the phenyl-substituted **205a** in the desymmetrization of 2-arylpropane-1,3-diols, ligand **205a** proved to be suitable for 2-ethylpropane-1,3-diol (**204**). A three-step sequence then furnished enone **207**, which underwent diastereoselective aldol reaction with fragment **208** to give compound **209**. Alkyne **210**, prepared from **209** in six steps, underwent addition with fragment **211** to yield compound **212**. Four subsequent steps, including oxidation of propargylic alcohol and cyclization between the hydroxy group and ynone, provided compound **213** with a pyranone ring. Treatment of **213** with Me_3_SnOH hydrolyzed the methyl ester, and intramolecular Yamaguchi esterification then led to lactone **214**, which was transformed into (−)-18-*epi*-peloruside A (**215**) in four steps.

**Scheme 27:**
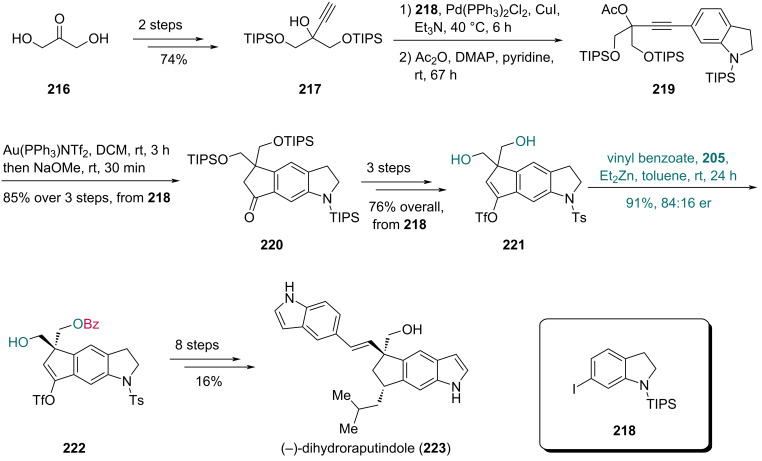
Lindel’s synthesis of (–)-dihydroraputindole (**223**).

In 2020, Lindel and co-workers reported their synthesis of (−)-dihydroraputindole D, featuring a Zn-catalyzed enantioselective benzoylation as the key step (Scheme 27) [69]. Using propargylic alcohol **217**, which was prepared from dihydroxyketone **216** in two steps, Sonogashira coupling with indoline **218** followed by acetylation afforded compound **219**. A Au-catalyzed cyclization and subsequent saponification with NaOMe gave indoline **220**. Three subsequent steps yielded diol **221**, which was treated with vinyl benzoate and a Zn-complex derived from Et_2_Zn and phenol **205** to afford benzoate **222** in 91% yield with 84:16 er. Finally, an eight-step sequence provided (−)-dihydroraputindole D (**223**).

### Local desymmetrization

Apart from enzymatic and transition-metal-catalyzed desymmetrization reactions, compounds with specific structures might also enable the desymmetrization by discriminating prochiral 1,3-diols in a diastereotopic manner. This strategy is termed as “local desymmetrization” [19,70].

**Scheme 28:**
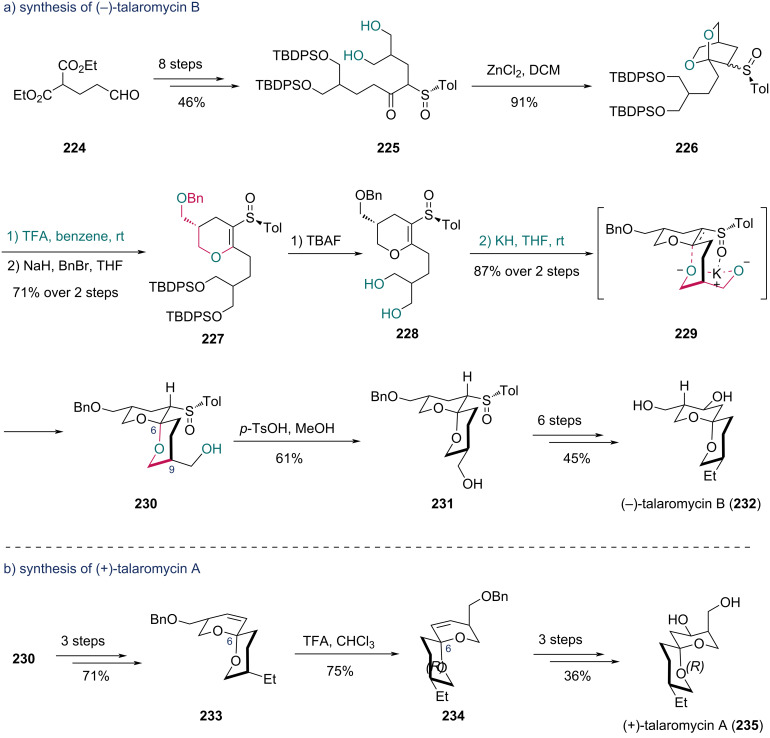
Iwata’s synthesis of a) (−)-talaromycin B (**232**) and b) (+)-talaromycin A (**235**).

In 1987, Iwata and co-workers completed the synthesis of (−)-talaromycin B and (+)-talaromycin A, two toxic metabolites from the fungus *Talaromyces stipitatus*, featuring asymmetric induction to forge chiral centers using a chiral sulfinyl group [71]. With their previously reported strategy [72], the chiral sulfinyl-containing diol **225** was prepared from diester **224** in eight steps (Scheme 28a). Treatment of **225** with ZnCl_2_ afforded dioxabicyclic compound **226**. Regioselective hydrolysis of **226** with TFA yielded a dihydropyran intermediate, which was benzylated to deliver **227**. Desilylation of **227** gave diol **228**, which underwent intramolecular Michael reaction to form bicyclic compound **230** as a single stereoisomer (87% yield over two steps). This scaffold with the desired C6 chiral center was constructed via intermediate **229**, where the sulfinyl group induced K^+^–oxygen chelation to form a six-membered transition state prior to protonation from the less hindered face. Acid-mediated epimerization at C9 of **230** yielded compound **231**, which was transformed into (−)-talaromycin B (**232**) in six steps. For (+)-talaromycin A (**235**) (Scheme 28b), a three-step transformation of **230** gave **233**, and subsequent isomerization at the C6 spirocenter with TFA produced compound **234**, which was converted into **235** in three additional steps.

**Scheme 29:**
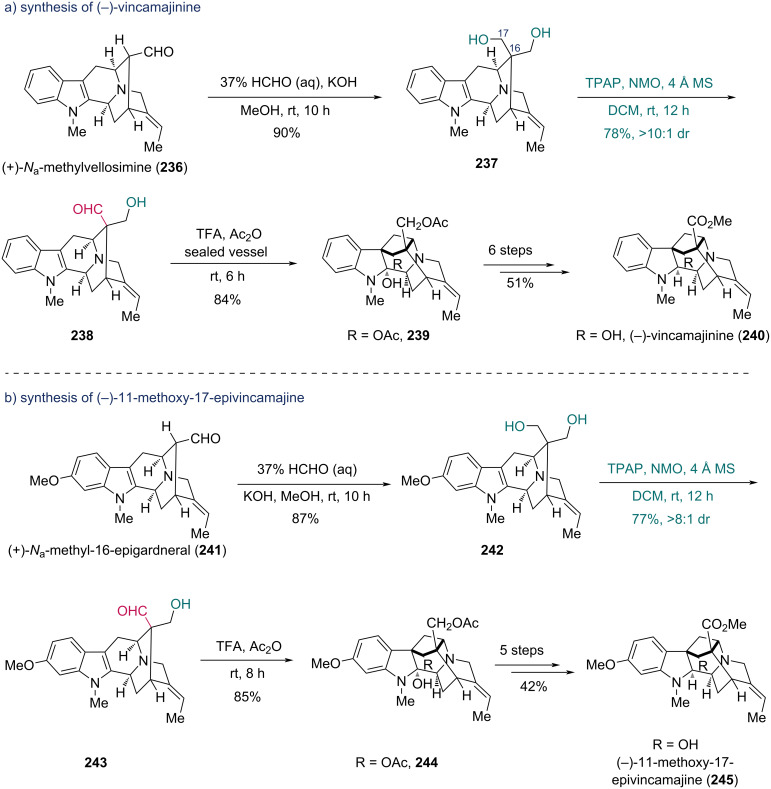
Cook’s synthesis of a) (−)-vincamajinine (**240**) and b) (−)-11-methoxy-17-epivincamajine (**245**).

The introduction of an inducing group such as a chiral sulfinyl group is effective in local desymmetrization, while substrates bearing caged frameworks and multiple chiral centers can also realize the desymmetrization. The Cook group reported the first total synthesis of (−)-vincamajinine and (−)-11-methoxy-17-epivincamajine, featuring a stereospecific cyclization as the key step (Scheme 29a) [73–74]. To obtain the cyclization precursor **238**, the prochiral diol **237** was prepared from (+)-*N*_a_-methylvellosimine (**236**) via a Tollens reaction. Subsequently, regioselective Ley–Griffith oxidation of **237** selectively targeted the C17 hydroxy group, affording aldehyde **238** in 78% yield with >10:1 dr. The high diastereoselectivity observed in the oxidation of the 1,3-diol indicated that the complex structure of the substrate could provide an environment of desymmetrization. The stereospecific cyclization of **238** was performed with trifluoroacetic acid (TFA) and Ac_2_O, along with acetylation of the free hydroxy group, to deliver compound **239** in high yield. A further six-step sequence completed the synthesis of (−)-vincamajinine (**240**). With the same strategy, (−)-11-methoxy-17-epivincamajine (**245**) was prepared from (+)-*N*_a_-methyl-16-epigardneral (**241**) (Scheme 29b). The synthesis of **244** was achieved through a similar sequence of steps: Tollens reaction of **241**, regioselective oxidation of diol **242**, and acidic cyclization of aldehyde **243**. Compound **244** was then converted into **245** in five additional steps.

**Scheme 30:**
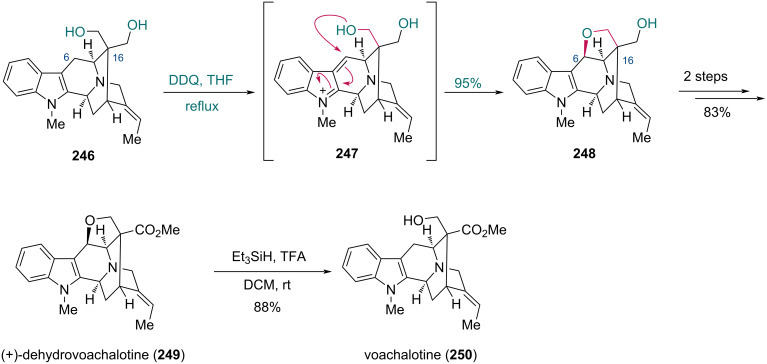
Cook’s synthesis of (+)-dehydrovoachalotine (**249**) and voachalotine (**250**).

The benzylic oxidative cyclization of indole derivatives mediated by 2,3-dichloro-5,6-dicyano-1,4-benzoquinone (DDQ) is an efficient strategy that the Cook group utilized in the total synthesis of several indole alkaloids [75–77]. In 2005, they reported the synthesis of vincamajine-related indole alkaloids, among which (+)-dehydrovoachalotine was prepared by a selective oxidative cyclization of a 1,3-diol moiety (Scheme 30) [74]. Treatment of the known prochiral diol **246** with DDQ first oxidized the benzylic C6 position to give intermediate **247**, followed by intramolecular attack of the hydroxy group to construct the tetrahydrofuran ring of compound **248**, establishing an expected C6 stereocenter and a chiral quaternary carbon center at C16. This desymmetrization was enabled due to the structural features of diol **246**, wherein the proximal hydroxy group was functionalized, while the distal hydroxy group remained intact. The synthesis of (+)-dehydrovoachalotine (**249**) was completed in two steps from **248**. Voachalotine (**250**) was further prepared from **249** in the presence of Et_3_SiH and TFA [78].

**Scheme 31:**
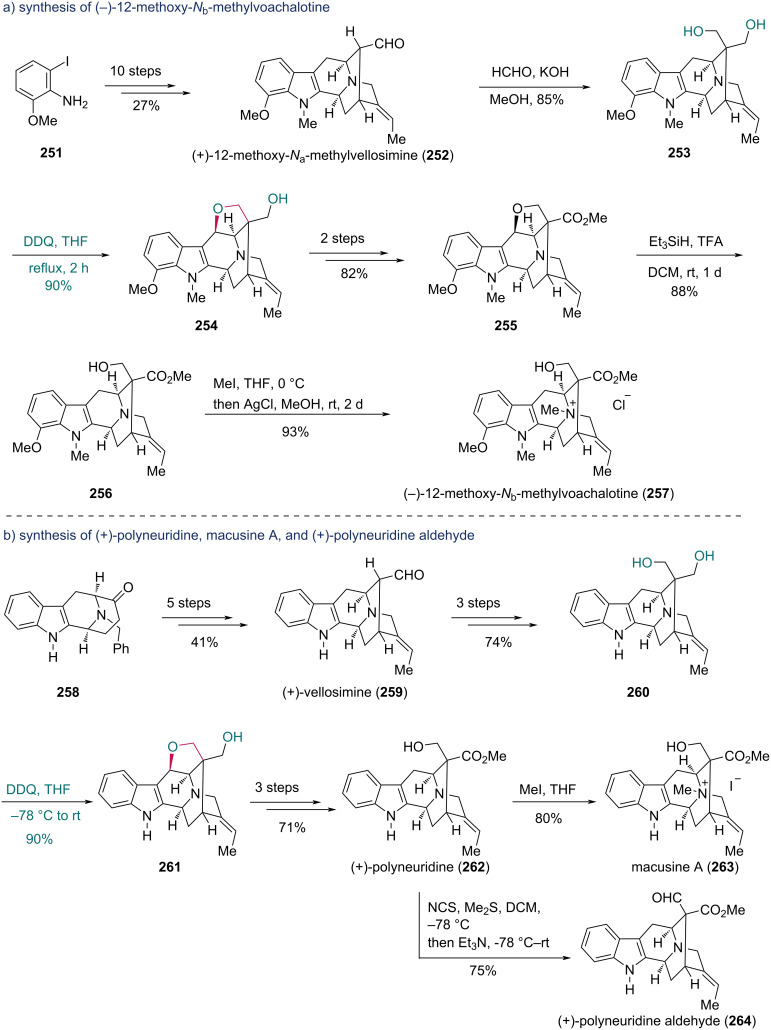
Cook’s synthesis of a) (−)-12-methoxy-*N*_b_-methylvoachalotine (**257**) and b) (+)-polyneuridine, macusine A, and (+)-polyneuridine aldehyde (**264**).

Using the same strategy, the Cook group synthesized (−)-12-methoxy-*N*_b_-methylvoachalotine, (+)-polyneuridine, (+)-polyneuridine aldehyde, and macusine A. In the synthesis of (−)-12-methoxy-*N*_b_-methylvoachalotine (Scheme 31a) [78], (+)-12-methoxy-*N*_a_-methylvellosimine (**252**) was first prepared in ten steps from aniline **251**, including a Larock indolization, Pictet–Spengler reaction, and Pd-catalyzed intramolecular cyclization. Tollens reaction of **252** gave diol **253**, which underwent DDQ-mediated oxidative cyclization to yield compound **254**. After a two-step conversion, the resulting compound **255** underwent reductive cleavage of the tetrahydrofuran ring with Et_3_SiH/TFA, giving compound **256**. Exposure of **256** to MeI in THF provided the corresponding *N*_b_-methiodide salt, which was subsequently converted into (−)-12-methoxy-*N*_b_-methylvoachalotine (**257**) upon treatment with AgCl in 93% yield. For the synthesis of (+)-polyneuridine, macusine A, and (+)-polyneuridine aldehyde (Scheme 31b) [79], (+)-polyneuridine (**262**) was first prepared as the common intermediate for macusine A and (+)-polyneuridine aldehyde. From compound **258**, vellosimine (**259**) was synthesized in five steps and subsequently converted into diol **260** in three steps. Oxidative cyclization of **260** with DDQ afforded compound **261**, which was further transformed into **262** in three steps. Finally, macusine A (**263**) was prepared by methylation of **262** with MeI, while (+)-polyneuridine aldehyde (**264**) was synthesized directly from alcohol **262** via Corey–Kim oxidation.

**Scheme 32:**
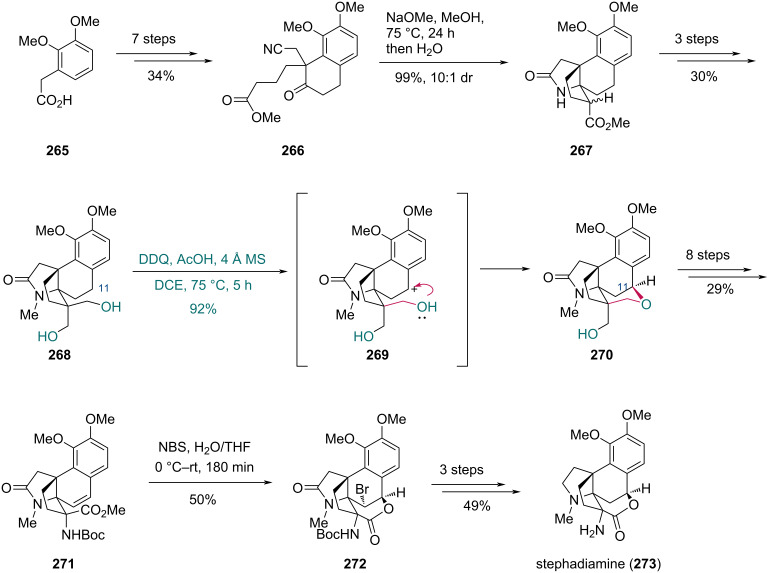
Trauner’s synthesis of stephadiamine (**273**).

The Trauner group also employed a similar strategy in the synthesis of stephadiamine in 2018 (Scheme 32) [80]. Starting from carboxylic acid **265**, compound **266** was prepared in a seven-step sequence. Then, the cascade cyclization was accomplished by treatment with NaOMe in MeOH, followed by H_2_O, affording compound **267** in excellent yield and diastereoselectivity. A subsequent three-step sequence gave diol **268**. Under DDQ and AcOH conditions, the benzylic C11 position of **268** was first oxidized to generate intermediate **269**, followed by intramolecular nucleophilic attack of the hydroxy group. This stereoselective cyclization constructed the tetrahydropyran ring of pentacyclic compound **270** in 92% yield and established the stereocenter at the C7 position. Compound **271**, prepared from **270** in eight steps, was treated with *N*-bromosuccinimide (NBS) in a H_2_O/THF solution to afford lactone **272** in 50% yield. Finally, **272** was converted to stephadiamine (**273**) in three steps.

**Scheme 33:**
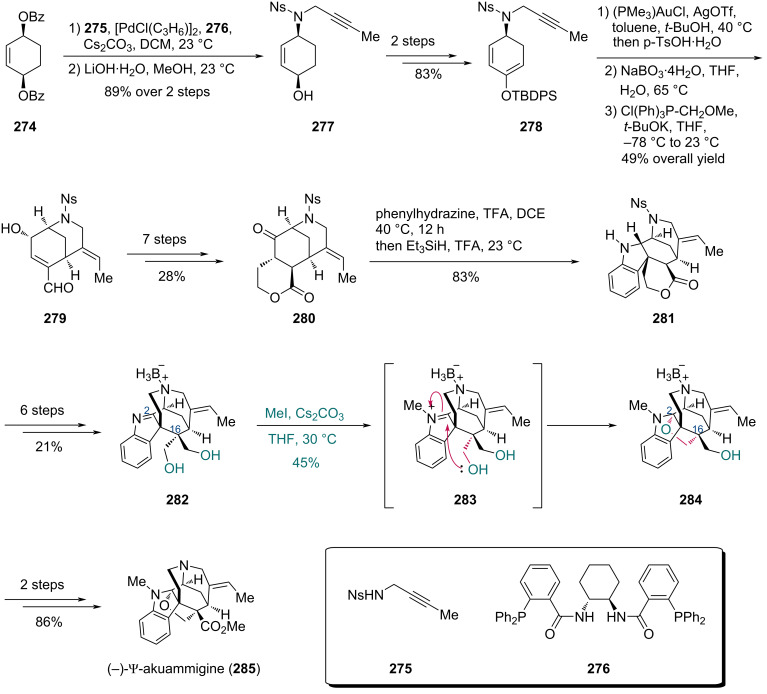
Garg’s synthesis of (–)-ψ-akuammigine (**285**).

In 2018, the Garg group completed the total synthesis of akuammiline alkaloids, including (−)-ψ-akuammigine (Scheme 33) [81]. The synthesis commenced with dibenzoate **274**, which underwent a Pd-catalyzed Trost desymmetrization using sulfonamide **275** and ligand **276**. Deprotection of the resulting adduct furnished alcohol **277**, which was subsequently converted to silyl enol ether **278** in two steps. Treatment of **278** with (PMe_3_)AuCl and AgOTf, followed by *p*-TsOH·H_2_O, effected a Au-catalyzed cyclization to construct the bicyclic core. This intermediate was then transformed into enal **279** via epoxidation and Wittig olefination. Seven additional steps converted enal **279** to lactone **280**, which then underwent a reductive interrupted Fisher indolization with phenylhydrazine to give indoline **281**. To forge the C16 stereocenter and form the C–O bond at C2, diol **282** was prepared from **281** in six steps. Treatment of **282** with MeI/Cs_2_CO_3_ induced cyclization through putative indoleninium intermediate **283**, wherein one hydroxy group underwent nucleophilic attack on the C2 electrophilic center while the other remained unreacted, giving furoindoline **284** in 45% yield. A final two-step transformation completed the synthesis of (−)-ψ-akuammigine (**285**).

**Scheme 34:**
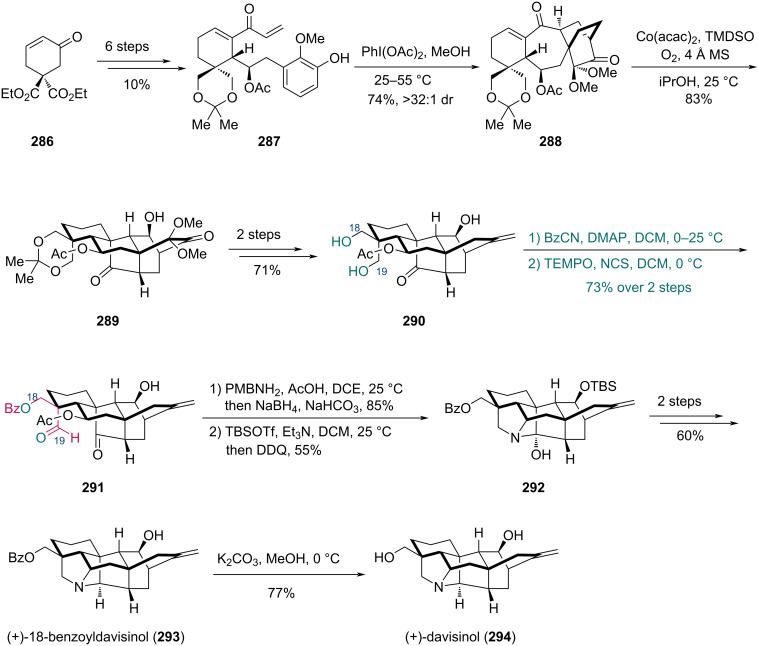
Ding’s synthesis of (+)-18-benzoyldavisinol (**293**) and (+)-davisinol (**294**).

In 2021, the Ding group reported the total synthesis of two hetisine-type diterpenoids (+)-18-benzoyldavisinol and (+)-davisinol [82] (Scheme 34). Using diester **286** as a starting material, phenol **287** was prepared in six steps. Subsequent oxidative dearomatization-induced Diels–Alder cycloaddition with PhI(OAc)_2_, delivered *endo*-cycloadduct **288** with high diastereoselectivity. Compound **288** was then treated with Co(acac)_2_, 1,1,3,3-tetramethyldisiloxane (TMDSO), and O_2_ in degassed iPrOH, undergoing a hydrogen-atom-transfer (HAT)-initiated redox radical cascade to give pentacyclic alcohol **289**, which was converted to C18/19 diol **290** in two steps. To differentiate the two hydroxy groups, the C18-alcohol was selectively protected by benzoylation using BzCN and 4-(dimethylamino)pyridine (DMAP) conditions, while the C19-alcohol was oxidized by TEMPO and *N*-chlorosuccinimide (NCS) subsequently. This two-step sequence provided ketoaldehyde **291** in 73% yield, demonstrating excellent site selectivity during the Bz protection. The assembly of the azabicyclic core was achieved in two steps from **291** via reductive amination followed by oxidative removal of the *p*-methoxybenzyl (PMB) group, giving heptacyclic compound **292**. Finally, (+)-18-benzoyldavisinol (**293**) was synthesized in two steps and subsequently deprotected to afford (+)-davisinol (**294**).

## Conclusion

In conclusion, over the past few decades, the enantioselective desymmetrization of prochiral 1,3-diols has become an important tool for constructing chiral centers and applied in various total syntheses. Several strategies, including enzymatic acylation, transition-metal-catalyzed acylation, and local desymmetrization have been adopted by chemists to synthesize complex molecules. A general survey of these examples revealed that enzymatic acylations using lipases (such as PPL, CAL, CRL and those from the *Pseudomonas* genus) are generally operated in mild conditions achieving relatively high yield and enantioselectivity. However, due to the intrinsic structural limitations of lipases, accessing the desired enantiomer requires laborious screening of enzymes. In the case of transition-metal-catalyzed acylations, the enantioselective desymmetrization of prochiral 1,3-diols within complex structures can be realized using organometallic catalysts composed of copper or zinc salts and different types of chiral ligands. In general, the ability to control the stereoselectivity of the product by using the enantiomer of the ligand in transition-metal-catalyzed acylations is a notable advantage compared to enzymatic methods. In the case of local desymmetrization, the enantioselectivity of the reaction depends predominantly on the inherent properties of the substrate.

Although numerous examples of enantioselective desymmetrization reactions of prochiral 1,3-diols via metal-catalyzed and enzymatic methods have been reported, these transformations are mostly limited to the acylation of hydroxy groups. Other reaction types, such as sulfonylation, oxidation, and coupling, remain underdeveloped in this context, suggesting significant progress is still needed in the methodological development for the enantioselective desymmetrization of prochiral 1,3-diols.

## Data Availability

Data sharing is not applicable as no new data was generated or analyzed in this study.
